# Partial Response to Sintilimab-Based Multimodal Therapy in a Refractory Primary Mediastinal Yolk Sac Tumor: A Case Report

**DOI:** 10.7759/cureus.94485

**Published:** 2025-10-13

**Authors:** Yibo Sun, Xue Li, Miaomiao Yang, Chunyu He

**Affiliations:** 1 Radiation Oncology, The Affiliated Cancer Hospital of Zhengzhou University and Henan Cancer Hospital, Zhengzhou, CHN

**Keywords:** alpha-fetoprotein, immunotherapy, multimodal therapy, primary mediastinal yolk sac tumor, sintilimab

## Abstract

Primary mediastinal yolk sac tumor (PMYST) represents an extremely rare and highly aggressive germ cell malignancy with poor prognosis and limited therapeutic options. We report a 55-year-old male who presented with a large anterior mediastinal mass measuring 149 × 73 mm and significantly elevated serum alpha-fetoprotein (AFP) levels (>1210 ng/mL). Histopathological examination and immunohistochemical staining confirmed the diagnosis of PMYST. An interim response was observed after one cycle of etoposide-cisplatin chemotherapy given concurrently with radiotherapy. However, upon completion of the entire chemoradiotherapy course (radiotherapy plus three cycles of chemotherapy), the patient was found to have disease progression with an enlarging tumor burden. Subsequently, sintilimab-based chemo-immunotherapy was initiated, resulting in a partial response achieved after four cycles of treatment. At the most recent follow-up, the patient demonstrates sustained clinical stability with continued disease control. This case highlights the potential efficacy of PD-1 inhibitor-based combination therapy in refractory mediastinal yolk sac tumor (YST), which may provide a novel therapeutic strategy for managing this challenging malignancy.

## Introduction

Primary mediastinal yolk sac tumor (PMYST) represents an exceptionally rare and highly aggressive subtype of germ cell neoplasm, predominantly affecting children and young adults [1,2], with a median age at presentation ranging from 23 to 28 years [3,4]. The pathogenesis is thought to involve aberrant migration of primordial germ cells during embryonic development [5]. These tumors primarily arise in the anterior mediastinum and commonly metastasize to the brain, lungs, liver, and bones, contributing to the overall poor prognosis [6]. Patients typically present with symptoms from local mass effect, such as chest pain, cough, dyspnea, or superior vena cava syndrome [6,7]. The diagnostic workup hinges on radiologic imaging, serum tumor marker assessment, and histologic confirmation. Histopathological examination remains the gold standard for diagnosis. Alpha-fetoprotein (AFP), secreted by yolk sac tumor (YST) cells, is a crucial biomarker for both diagnosis and prognosis, with approximately 80% of patients exhibiting elevated AFP levels [7,8]. Immunohistochemical analysis of biopsy specimens typically demonstrates positive staining for AFP, Glypican-3, SALL4, and placental alkaline phosphatase (PLAP) [9], which is crucial for differentiating PMYST from other anterior mediastinal malignancies, including thymoma, lymphoma, and other germ cell tumors [3,10]. Mediastinal germ cell tumors exhibit significantly more aggressive behavior and carry a substantially worse prognosis compared to their gonadal counterparts [10].

The commonly used treatment strategy involves platinum-based chemotherapy followed by surgical resection of residual masses [5]. Successful treatment with radiotherapy has occasionally been reported [11,12]. However, refractory cases pose considerable therapeutic challenges. Herein, we report a case of primary anterior mediastinal yolk sac tumor that achieved sustained partial response following combination treatment with platinum-based chemotherapy, radiotherapy, and the PD-1 inhibitor sintilimab, highlighting the potential role of immunotherapy in managing this challenging malignancy.

## Case presentation

**Figure 1:**
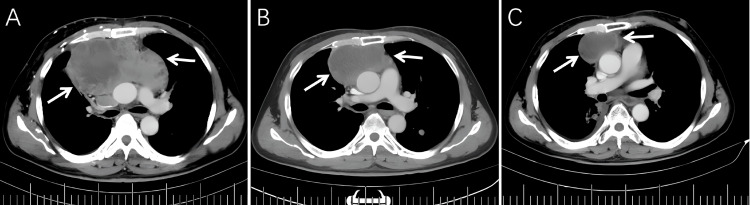
Serial chest CT images (mediastinal windows) demonstrating temporal changes in the anterior mediastinal mass. (A) The thorax CT scan obtained on the first admission showed a mass in the anterior mediastinum with the size of 149 × 73 mm. (B) After chemoradiotherapy, the tumor has decreased in size (94 × 57 mm). (C) Subsequent CT examination after four cycles of combined immunotherapy (sintilimab) with chemotherapy demonstrates further substantial tumor regression, measuring 62 × 42 mm. (White arrows indicate the anterior mediastinal mass.)

A 55-year-old male presented on February 2, 2023, with a two-month history of progressive chest tightness and facial swelling. Physical examination revealed facial edema and distended neck veins, suggestive of superior vena cava (SVC) syndrome. Initial imaging and biopsy at an external hospital revealed a large anterior mediastinal mass and a diagnosis of poorly differentiated carcinoma. Given the clinical presentation of SVC syndrome and the locally advanced, inoperable nature of the disease, empirical chemotherapy with docetaxel and cisplatin was initiated. However, following two cycles, no appreciable symptomatic or radiological improvement was observed. Contrast-enhanced computed tomography (CT) of the chest revealed a large, irregularly shaped soft tissue mass (149 × 73 mm) in the anterior mediastinum, exhibiting heterogeneous enhancement (Figure 1A).

**Figure 2:**
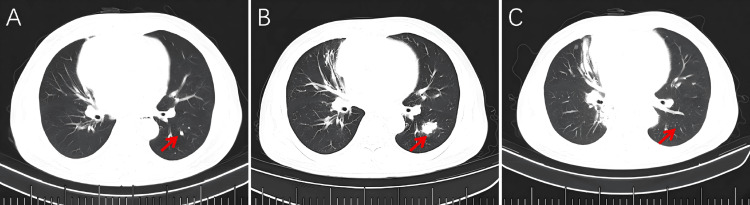
Serial chest CT images (lung windows) demonstrating the interval change in pulmonary nodules. (A) Initial CT scan demonstrates scattered small pulmonary nodules in both lung fields. (B) After chemoradiotherapy, the pulmonary nodules increased in size. (C) CT scan obtained after four cycles of combined immunotherapy (sintilimab) with chemotherapy shows an interval decrease in both the size and number of previously identified pulmonary nodules when compared to the prior study. (Red arrows indicate the pulmonary nodules.)

Laboratory tests showed markedly elevated tumor markers: alpha-fetoprotein (AFP) >1210 ng/mL (reference level <10 ng/mL), carcinoembryonic antigen (CEA) 15.32 ng/mL (reference level <5 ng/mL), and beta-human chorionic gonadotropin (β-HCG) <0.10 mIU/mL (reference level <1 mIU/mL). An ultrasound-guided core needle biopsy of the mediastinal mass revealed malignant epithelial cells with extensive necrosis. Immunohistochemical analysis showed positive staining for cytokeratin (CK), SALL4, AFP, CK8/18, BRG1, and Glypican-3, with a high Ki-67 proliferation index of 90%. Staging evaluation with contrast-enhanced computed tomography (CT) of the neck, chest, and abdomen was performed to assess the extent of disease. Besides the large anterior mediastinal mass and the previously noted pulmonary nodules (Figure 2A), no other distant metastatic sites were identified. No primary tumor was detected in the gonads or other locations. Combined with the immunohistochemical profile and significantly elevated serum AFP, these findings confirmed the diagnosis of primary anterior mediastinal yolk sac tumor. PD-L1 protein testing: PD-L1 (22C3) CPS = 2, PD-L1 (SP263) CPS = 2. 

Following multidisciplinary consultation for a surgically inoperable bulky mediastinal mass with vascular invasion and suboptimal response to two chemotherapy cycles, palliative IMRT (50 Gy/25 fx) was administered from February 6 to March 16, 2023. Due to concerns of bleomycin-induced pulmonary toxicity, the chemotherapy regimen was switched to EP (etoposide + cisplatin). The first cycle of EP was administered concurrently with radiotherapy from February 20. An interim response assessment was performed on March 4, 2023, during the radiotherapy course, which demonstrated modest tumor regression (139 × 73 mm) and a significant decrease in serum tumor markers, indicating a positive response to the ongoing concurrent chemoradiation. Following the completion of radiotherapy, the patient received two additional sequential cycles of EP chemotherapy on April 8 and May 12, 2023, to consolidate the response.

A definitive restaging was performed after the completion of all planned therapy on June 15, 2023. This CT scan revealed disease progression with enlargement of existing pulmonary nodules and the emergence of new ones (Figures 1B, 2B), indicating metastatic relapse. As a result, combination chemo-immunotherapy with cisplatin, etoposide, and sintilimab was initiated on June 21, 2023.

Restaging CT on July 20, 2023, showed a significant reduction in both mediastinal and pulmonary lesions, with the primary mass measuring 77 × 56 mm. The patient then received three additional cycles of the same regimen on July 24, August 30, and September 27, 2023. During this treatment period, the patient developed mild pneumonitis, which resolved with prophylactic antibiotic therapy. Follow-up CT on September 25, 2023, showed further tumor regression (62 × 42 mm) (Figure 1C). Pulmonary nodules show an interval decrease in size or complete resolution compared to previous studies (Figure 2C). By October 1, 2023, serum AFP normalized to 1.26 ng/mL. The patient subsequently received four cycles of sintilimab maintenance therapy. Subsequent follow-up CT scans were performed every two months, with the latest scan on March 5, 2024, showing a mediastinal mass measuring 49 × 22 mm. The patient remains under active surveillance, demonstrating sustained disease control and excellent performance status.

## Discussion

In the present case, the patient's disease course highlights the considerable therapeutic challenges in managing refractory PMYST. Due to the rarity of this malignancy, no standardized treatment protocols have been established [13]. Chemotherapy remains the cornerstone of treatment, with platinum-based regimens such as bleomycin, etoposide, and cisplatin (BEP), or vinblastine, ifosfamide, and cisplatin (VIP), being the preferred first-line options. Typically, a minimum of four cycles is recommended, with VIP favored for its more favorable toxicity profile [14]. According to the International Germ Cell Cancer Collaborative Group, the five-year progression-free survival rate for YST is only 35%, and it drops below 50% in cases with distant metastases [15,16]. Although most tumors initially respond to chemotherapy, they often exhibit inherent resistance to radiotherapy [11]. Some studies suggest that radiotherapy following neoadjuvant chemotherapy may improve local control by enhancing radiosensitization [12].

In our patient, initial chemoradiotherapy showed limited efficacy, with pulmonary metastases developing after three cycles of treatment. The subsequent introduction of sintilimab-based chemo-immunotherapy resulted in significant tumor regression and a marked reduction in serum AFP levels. These results suggest that not only does the PD-1 inhibitor play a role, but the prior radiotherapy may also have contributed by modulating the tumor immune microenvironment, thereby creating favorable conditions for the subsequent immunotherapy [17]. Similar findings have been reported in the literature, indicating that combining immune checkpoint inhibitors with chemotherapy may be particularly effective in metastatic settings [14]. PD-L1 expression, though low in this mediastinal yolk sac tumor, is recognized in germ cell tumors [18], supporting immune checkpoint inhibition.

## Conclusions

PMYST is a rare and highly aggressive malignancy with a poor prognosis. A multidisciplinary treatment approach, incorporating the potential synergy between local modalities like radiotherapy and systemic immunotherapy, particularly one combining chemotherapy with immunotherapy, may improve clinical outcomes. Treatment strategies should be individualized based on histopathological features and metastatic status, with close monitoring of AFP levels and serial imaging to guide therapeutic decisions. Personalized treatment approaches are especially important in cases with atypical disease progression, such as pulmonary metastasis.
